# Increasing Diversity, Equity, and Inclusion in the Health and Health Services Research Workforce: A Systematic Scoping Review

**DOI:** 10.1007/s11606-024-09041-w

**Published:** 2024-09-25

**Authors:** Margaret W. Gichane, Ida Griesemer, Leah Cubanski, Blessing Egbuogu, D. Keith McInnes, Lynn A. Garvin

**Affiliations:** 1 Department of Obstetrics, Gynecology & Reproductive Sciences, University of California, San Francisco, CA USA; 2 White River Junction VA Medical Center, Hartford, VT USA; 3 Georgetown University Law Center, Washington, D.C. USA; 4 U.S. Department of Veterans Affairs, HSR&D Center for Healthcare Organization and Implementation Research, Washington, D.C. USA; 5 VA Bedford Healthcare System, Bedford, MA USA; 6 Department of Health Law, Policy and Management, Boston University School of Public Health, Boston, MA USA; 7 VA Boston Healthcare System, Boston, MA USA

**Keywords:** diversity, equity, and inclusion, health services research workforce, recruitment, promotion, retention

## Abstract

Health and health services research institutions seek to increase diversity, equity, and inclusion (DEI) to overcome structural bias. The objective of this review is to identify, characterize, and evaluate programs aimed to strengthen DEI in the health and health services research workforces. We conducted a systematic scoping review of literature of 2012–2022 North American peer-reviewed empirical studies in PubMed and Embase using the Arksey and O’Malley approach. This review identified 62 programs that varied in focus, characteristics, and outcomes. Programs focused on supporting a spectrum of underrepresented groups based on race/ethnicity, gender identity, sexual orientation, disability status, and socioeconomic status. The majority of programs targeted faculty/investigators, compared to other workforce roles. Most programs were 1 year in length or less. The practices employed within programs included skills building, mentoring, and facilitating the development of social networks. To support program infrastructure, key strategies included supportive leadership, inclusive climate, resource allocation, and community engagement. Most programs evaluated success based on shorter-term metrics such as the number of grants submitted and manuscripts published. Relatively few programs collected long-term outcomes on workforce pathway outcomes including hiring, promotion, and retention. This systematic scoping review outlined prevalent practices to advance DEI in the health and health services research field. As DEI programs proliferate, more work is needed by research universities, institutes, and funders to realign institutional culture and structures, expand resources, advance measurement, and increase opportunities for underrepresented groups at every career stage.

**Supplementary Information:**

The online version contains supplementary material available at 10.1007/s11606-024-09041-w.

## INTRODUCTION

Health and health services research institutions recognize the moral imperative as well as clinical and financial advantage of workforce diversity, equity, and inclusion (DEI): building workforces that reflect the *diversity* of the society they serve; ensuring the *equity* of policies, programs and outcomes; and practicing *inclusion* to help all members feel a sense of belonging.^1,2^ DEI initiatives focused on historically marginalized, underrepresented groups in the health and health services research workforce are expanding, but progress has been gradual. Current data show that Black/African Americans, Hispanic/Latinos, American Indian/Alaska Natives; women; people with disabilities; lesbian, gay, bisexual, transgender, and queer (LGBTQ+) individuals; and people from low socioeconomic backgrounds are underrepresented among doctoral degree holders in the biomedical and health sciences.^3^ Within health services, a national study found two-to-three times greater representation among Asian Americans compared to other races/ethnicities combined, yet lower representation among Black/African American and Hispanic individuals among the US health services research workforce and health services program graduates.^4,5^ Further, individuals from underrepresented groups also experience systemic inequities and exclusion within the workforce. A 2021 study found that medical faculty who were male, White, and tenured or tenure-eligible had higher promotion rates than faculty who were either female, an underrepresented racial/ethnic minority, or nontenured.^6^ Black principal investigators, particularly women, are less likely to be funded, as is research that specifically focuses on health inequities experienced by marginalized groups.^7^

Review articles in the past decade have documented the increase in health and health services research diversity efforts. An environmental scan of 124 US medical schools conducted by Adanga et al. (2012) found that only 36 (29%) had specific programs targeted to underrepresented (URM) faculty, and 68 (55%) did not evaluate program results.^8^ Other reviews have focused on mentoring of URM faculty. Beech et al. (2013) reviewed 18 US academic medical centers, recommending reduced reliance on external funding to increase program sustainability.^9^ Ransdell et al. (2021) reviewed 46 studies and recommended matching mentors and mentees based on personality, culture, goals, and expectations.^10^ Urena et al. (2021) identified the need for (1) culturally diverse and representative mentorship, (2) multi-stage career development, and (3) incorporation of theory into program design.^11^ Crites’ et al. (2022) review of 58 medical school faculty mentorship programs found that although 42% of faculty were women, only eight (14%) of programs targeted women.^12^ Similarly, Williams et al. (2022) determined that only three of 27 mentorship programs in academic medicine studied supported diversity or measured outcomes.^13^

To date, however, no reviews have examined a strategic variety of DEI initiatives, different health service research roles, i.e., faculty/investigators, postdoctoral scholars, medical residents and research staff, or steps in the workforce pathway, i.e., hiring, promotion and retention. Nor have reviews examined a spectrum of underrepresented researcher groups targeted by DEI programs, i.e., by race, ethnicity or religion, gender identity, sexual orientation, disability status, and socioeconomic status. Thus, is it incumbent upon health leaders and practitioners to highlight recent programs and practices that have measurably advanced DEI in the health and health services research field.

The objective of this systematic scoping review was to identify, characterize, and evaluate DEI program approaches aimed at accelerating the hiring, promotion, and retention of employees from underrepresented groups in the health and health services research workforce. Our aims were to (1) identify and describe the DEI program characteristics, goals, and success measurement and highlight any gaps; (2) distinguish DEI program leadership and infrastructure initiatives, and their challenges and opportunities; and (3) distinguish DEI program practices (e.g., mentoring, research skills training), and their challenges and opportunities.

## METHODS

### Search Strategy and Study Selection

This systematic scoping review was conducted in accordance with the Arksey and O’Malley approach^14^ and followed the Preferred Reporting Items for Systematic reviews and Meta-Analyses extension for Scoping Reviews (PRISMA-ScR) checklist.^15^ An *a priori* protocol of the review was registered at Open Science Framework (https://osf.io/temdc).

Focusing on contemporary programs and approaches, we conducted a search of the 2012–2021 literature on January 19, 2022, using two comprehensive electronic databases of general biomedical literature — PubMed and Embase^16,17^ — and updated the search on January 13, 2023, to identify additional articles published in 2022. Table 1 lists term definitions, inclusion and exclusion criteria, and rationale. Based on National Institutes of Health (NIH) Guidelines, we defined underrepresented groups to include underrepresented minority by race, ethnicity, or religion; those underrepresented by gender identity (women); sexual orientation (LGBTQ+); and disability status and lower socioeconomic status (SES).^18^ The initial search dates of 2012–2021 provided a view of the past decade’s DEI programs to keep findings timely; the addition of articles published in 2022 kept the findings up-to-date.

**Table 1:** Term Definitions, Inclusion and Exclusion Criteria, and Rationale for DEI Systematic Scoping Review

Term definitions
- “Underrepresented minorities” (URMs) include races or ethnicities whose representation in science, technology, engineering and medicine (STEM) employment and education is smaller than their representation in the US population, per the definition of the US National Science Foundation. This includes Blacks or African Americans, Hispanics or Latinos, and American Indians or Alaska Natives - “Underrepresented groups” include underrepresented minority (URM) by race, ethnicity, or religion; those underrepresented by gender identity (women); sexual orientation (LGBTQ+); disability status and lower socioeconomic status (SES), based on the *NIH-Wide Strategic Plan for Diversity, Equity, Inclusion and Access – Fiscal Years 2023–2027* - “Health and Health Services Research Workforce” includes faculty, non-administrative research staff, medical residents (if involved with research), and post-doctoral fellows, informed by Frogner et al. (2018; 2022). Excluded from this definition are groups who do not conduct grant-supported research: research study participants (subjects); students (e.g., high school, college, graduate or medical school); medical residents who are not doing research; and administrators - Title/abstract search terms were selected based on the key research questions, foundational literature on DEI initiatives in the HSR workforce addressed in our “Introduction,” with expert advice from the Chief Medical/Health Sciences Librarian for our research center. Terms of HSR Workforce searched: research, workforce or work force, employee, facult (word stem), staff, research assistant, apprentice. Terms of DEI searched: divers (word stem), equit (word stem), inclus (word stem), underrepresent, underserve, disparit (word stem), minorit (word stem), disab, race, racial, ethnic, religio (word stem), socioecon, socio-econ, gender, impair, sex, African American, Black, Hispan (word stem), Native, Hawaii, Pacific Islander, Asian American, Senior, Older, and NOT undergraduate nor high school. Please see Chart A “Search Terms and Strategy” (available as online supplement to this article at http://www.hsr.org).
Inclusion criteria and rationale
1. Peer-reviewed articles to focus on academic and institutional programs, acknowledging increased risk of publication bias 2. Published in the English language in the USA or Canada, as both HSR workforces might reflect similar though not identical DEI challenges and opportunities. Articles published January 2012 to December 2022 were chosen to keep evidence timely 3. Focus on any program, intervention, initiative, or practice designed to increase/sustain DEI in health and health services research workforce—from recognized practices, e.g., mentoring, to uncommon practices, e.g., biostatics support, to show the range 4. Support underrepresented groups (see Definition, above). 5. May address support to researchers at any career stage, e.g., recruitment, promotion, and retention 6. May address support to specified health and health services research workforce roles (see Definition, above)
Exclusion criteria and rationale
1. Literature reviews, systematic reviews, conceptual papers and discussion pieces, conference and poster abstracts, and news articles 2. Does not focus on a program, intervention, initiative, or practice implemented to increase and/or sustain health and health services research workforce diversity, i.e., studies that simply describe or evaluate workforce diversity are excluded 3. Does not specifically support underrepresented groups (see Definition, above)

The search strategy, terms, and database selection were determined in consultation with the chief librarian of a large academic medical center with expertise in medical and health services literature. References from four DEI reviews and an environmental scan were evaluated to identify additional articles that met our criteria.^8–11^

**Figure 1:**
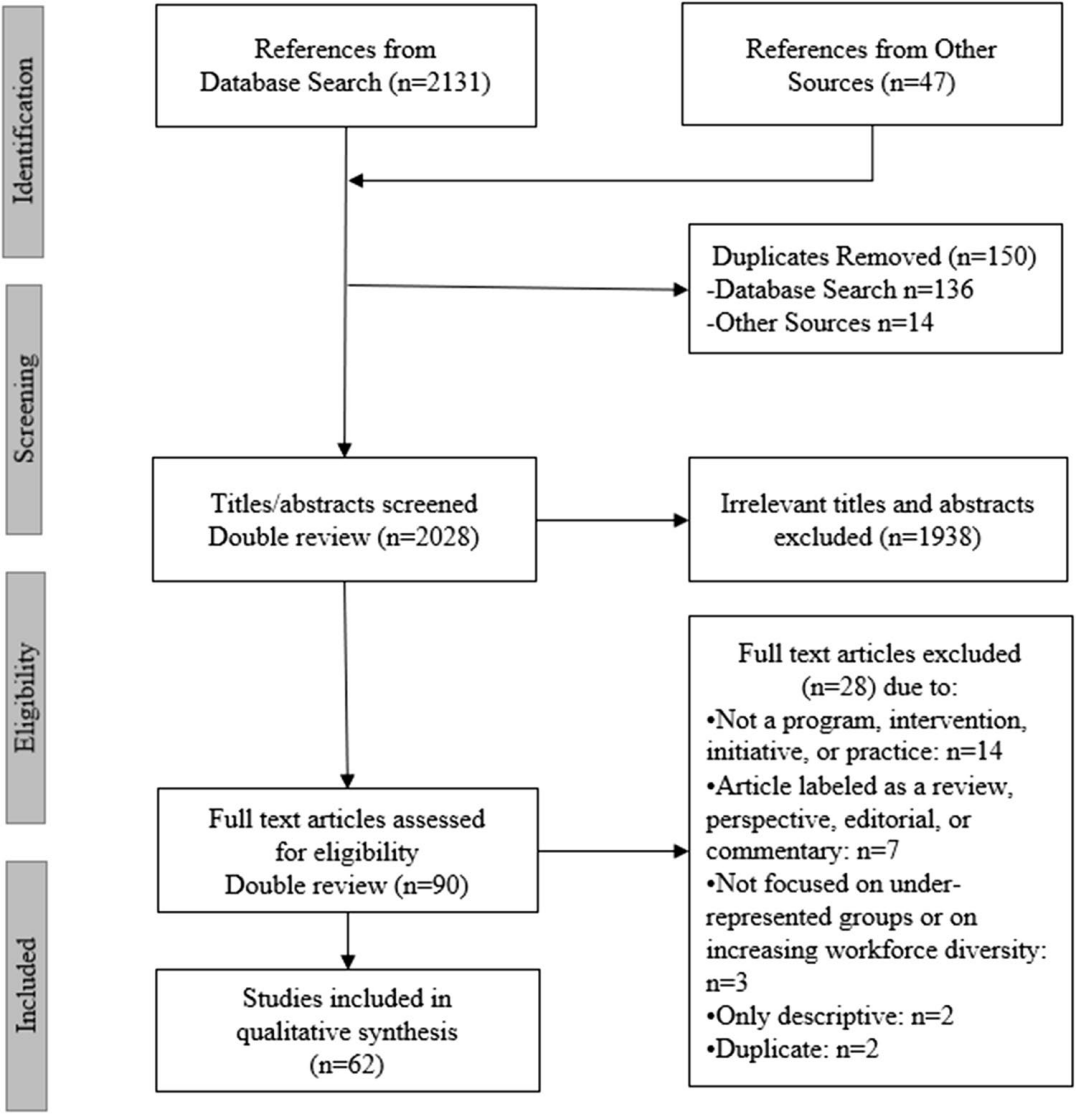
PRISMA-ScR flowchart of study selection.

Identical protocols were followed for reviewing 2012–2021 studies and 2022 studies. The four independent reviewers for the 2012–2021 studies were LG, MG, IG, and LC; in review of 2022 studies, BE replaced LC. Following each search, duplicates were removed. Citations identified were imported and stored in Covidence.^19^ Covidence was used for title and abstract screening and for full-text review. Before screening all articles, four reviewers (IG, LC, LG, MG) piloted the screening process on six articles, clarifying the eligibility criteria to assure consistent interpretation across reviewers.^20,21^ To screen the remaining titles and abstracts, one pair of independent reviewers (MG and IG) screened publications whose first author surnames started with A–M; the other pair (LG and LC for 2012–2021 studies; LG and BE for 2022 studies) screened publications whose first author surnames started with N–Z. Next, the full texts of selected citations were divided between the same two pairs of reviewers and assessed in detail against the inclusion and exclusion criteria. Reasons for exclusion of publications at full-text review were recorded and reported in Covidence. Any disagreements among reviewers throughout the selection process were resolved through consensus among all four reviewers. The results of the search and the study inclusion process were reported in the final scoping review and presented in the (PRISMA-ScR) flow diagram (Fig. 1).

### Data Collection and Synthesis

Data extraction was conducted using categories based on the research questions. Categories included these program elements: (1) institution and program name; (2) program geography, length, and article’s analysis timeframe; (3) program aims and (4) approaches (e.g., mentoring, didactic training); (5) program focus on workforce role (e.g., staff, faculty); (6) program focus on underrepresented groups (e.g., race/ethnicity, gender); (7) program challenges and opportunities; and (8) program outcomes, if reported. Data were extracted by the two pairs of independent reviewers, each pair addressing half of the studies. Extracted information was divided into Table 2, Appendix B and C. Finally, we used rapid qualitative inquiry^22^ to build a summarizing framework for all 62 studies.

**Table 2:** Characteristics and Measures of DEI Programs in Health and Health Services Research

		Leadership and infrastructure			Practices		
Program characteristics and success measures	Total	Leader-ship/culture	Funds/re-sources/implement	Commu-nity engage	Mentor-ing	Research skills training	Social network
Article total	*N*=62 (%)	*N*=10 (%)	*N*=23 (%)	*N*=7 (%)	*N*=49 (%)	*N*=62 (%)	*N*=11 (%)
Program length (1 h–5 years)							
1–70 h	5 (8)	1 (10)	--	2 (29)	2 (4)	5 (8)	--
1–3 weeks	5 (8)	1 (10)	--	--	4 (8)	5 (8)	--
2–6 months^1^	6 (10)	1 (10)	1 (4)	--	5 (10)	6 (10)	2 (18)
1 year^2^	14 (21)	1 (10)	6 (26)	--	12 (24)	14 (21)	1 (9)
>1 year^3^	16 (27)	1 (10)	9 (39)	4 (57)	16 (33)	16 (27)	5 (45)
N/A or not specified	16 (27)	5 (50)	7 (30)	1 (14)	10 (20)	16 (27)	3 (27)
Recipients’ workforce role							
Faculty/investigator^4^	53 (84)	7 (70)	19(83)	3 (43)	43(88)	53 (84)	9 (82)
Postdoctoral/med resident^5^	20 (32)	4 (40)	7 (30)	3 (43)	15 (31)	20 (32)	3(27)
Research staff^6^	8 (13)	1 (10)	--	2 (29)	1 (2)	8 (13)	--
Underrepresented group served							
Race/ethnicity^7^	58 (92)	10(100)	22(96)	6 (86)	47 (96)	58 (92)	10 (91)
Gender^8^	15 (24)	2 (20)	6 (26)	2 (29)	12 (24)	15 (24)	3 (27)
Socioeconomic status^9^	8 (13)	--	3 (13)	2 (29)	6 (12)	8 (13)	1 (9)
Disability status^10^	7 (11)	1 (10)	1 (4)	--	5 (10)	7 (11)	1 (9)
Sexual orientation^11^	5 (8)	1 (10)	1 (4)	1 (14)	3 (6)	5 (8)	2 (18)
Not specified	1 (2)	-	1 (4)	--	-	1 (2)	--
Workforce pathway target							
Hiring/recruitment^12^	11 (18)	1 (10)	3 (13)	5 (71)	8 (16)	11 (18)	4 (36)
Tenure/promotion^13^	11 (18)	3 (30)	4 (17)	1 (14)	10 (20)	11 (18)	5 (45)
Retention^14^	8 (13)	2 (20)	4 (17)	1 (14)	6 (12)	8 (13)	1 (9)
Not specified	37 (60)	5 (50)	13 (57)	--	33 (67)	37 (60)	2 (18)
Program success measures							
Grants awarded^15^	35 (56)	6 (60)	16 (70)	5 (71)	32 (65)	35 (56)	8 (73)
Manuscripts/proceedings^16^	28 (45)	6 (60)	13 (57)	4 (57)	26 (53)	28 (45)	9 (82)
Recipients’ research skills^17^	8 (13)	2 (20)	--	2 (29)	7 (14)	8 (13)	1 (9)
Inclusive climate	4 (6)	--	2 (9)	--	3 (6)	4 (6)	--
Additional degrees earned	2 (3)	1 (10)	2 (9)	--	1 (2)	2 (3)	1 (9)
Mentorship quality	3 (5)	--	2 (9)	--	2 (4)	3 (5)	1 (9)
Teaching positions gained	1 (2)	--	1 (4)	--	1 (2)	1 (2)	1 (9)
Not specified	11 (18)	2 (20)	6(26)	1 (14)	7 (14)	11 (18)	--

Citations for program characteristics and measures

1. Length, 2–6 months: ^34,53,54,71,73,84^

2. Length, 1 year: ^24,36,37,43,45,48,50,53,69,76–79,81^

3. Length, >1 year: ^29,33,40,42,46,47,51,57,63,65,66,70,75,80,82,83,85^

4. Faculty/investigator: ^29,33,40,42,46,47,51,57,63,65,66,70,75,80,82,83,85^

5. Postdoctoral/Med resident: ^26,28,30,40,46,53,55,56,63,65,72,74,76^

6. Research staff: ^25,39,49,54,60,61^

7. Race/ethnicity: ^24,26–29,31–41,43–72,74–76,78,82,84^

8. Gender: ^33–37,39,40,62,67,68,70,72–75,78,82,84^

9. Socioeconomic status: ^25,33,37,46,56,62,67,74^

10. Disability status: ^29,37,39,41,46,50,56,67,68^

11. Sexual orientation: ^36,39,74,75,80^

12. Hiring/recruitment: ^24,25,31,35,36,44,46,61,62^

13. Tenure/promotion: ^28–30,34,41,66,67,78,80,81^

14. Retention: ^30,33,47,51,63,65,75,80,85^

15. Grants: ^24,28–31,33–37,41–43,45,47,48,50,55,57,60,63,65–68,71,73,75,78–80,83,85^

16. Manuscripts: ^25,29,34–37,41–45,47,50,55,57,59,63–65,67,71,75,78–80,82,85^

17. Research skills: ^25,29,34–37,41–45,47,50,55,57,59,63–65,67,71,75,78–80,82,85^

## RESULTS

We identified 2131 studies through search and 47 additional studies through reference harvesting (Fig. 1).^23^ After removing duplicates, we screened 2028 study titles and abstracts. Full-text examination of 90 articles resulted in 62 studies included in this systematic scoping review.^24–85^

Rapid qualitative inquiry^22^ distinguished six types of program initiatives in the literature — three that addressed *program leadership and infrastructure* (i.e., leadership and cultural climate; funding, resources and implementation; and community engagement), and three focused on *program practices* (i.e., mentoring; research skills development; and social networking). We note that the majority of DEI programs reviewed combined multiple practices and infrastructure elements.

Characteristics and success metrics of the 62 studies are catalogued in Appendix A. All articles featured American-based programs; none from Canada was identified. Fully 61 programs were funded by the NIH; only one was funded by the US Centers for Disease Control and Prevention (CDC).^75^

Among the 46 programs specifying length, most were 1 year or longer; the other programs ranged from 1 h to 6 months (Table 2). Approximately 84% of DEI programs sought to increase diversity among faculty or investigators. Some programs also targeted postdoctoral scholars or medical residents in research, but relatively few targeted research staff. Nine out of every ten studies focused on supporting individuals from underrepresented groups based on race/ethnicity, while 24% focused on underrepresented groups based on gender. Few programs specifically named support for researchers underrepresented based on SES, disability status, and sexual orientation. Forty percent of articles tracked success in hiring, promotion, and retention. Fifty-six reported success in number of grants awarded to recipients. Other success metrics included number of manuscripts or conference proceedings produced and research skills acquired.

### DEI Program Characteristics, Challenges, and Opportunities

#### DEI Program Leadership and Infrastructure

Three program strategies for building DEI infrastructure comprised (1) supportive leadership and an inclusive climate,^86^ (2) increased funding and resources, and (3) community engagement. The most evident gap among leadership and infrastructure strategies was the lack of institution-level, longer term goals and success measures for program scope and sustainability.

### Leadership and Cultural Climate

The ten articles address leadership^28,30,34,64,75^ and cultural climate.^31,39,49,54,58^ Programs described the importance of having leadership that is supportive of DEI at all levels to signal the organization’s long-term vision and commitment, and to align resources and systems.^28,30,75^ At the national level, programs were often born out of the prioritization of DEI within the organization’s strategic planning,^28,64^ highlighting the need for advocacy among organization membership. At the institutional level, programs reported on the role of DEI leadership committees tasked with guiding a broad spectrum of policies, practices, and activities.^64^ Programs also emphasized building leadership capacity among their underrepresented group participants.^30,75^

Strategies to create an inclusive climate largely centered around providing trainings. These trainings focused on unconscious bias,^49^ addressing problematic behaviors in the workforce (e.g., bias and bullying)^39^ and inclusive excellence.^54,58^ For example, the *Black Voices in Research* curriculum aimed to provide “instructional materials that showcase inclusive excellence, facilitate the dialog about diversity and inclusion in biomedical research, enhance critical thinking and reflection, integrate diverse visions and worldviews, and ignite action.” Two articles described more comprehensive institutional approaches to creating an inclusive climate. Mount Sinai described a double-pronged approach focused on increasing the presence of women and individuals from underrepresented racial/ethnic groups through pathway programs and programs for faculty trainees, as well as development of DEI-focused committees.^31^ Mayo Clinic’s approach focused on integrating inclusion into recruitment, using training and feedback to improve workforce culture, and providing assessments and support to increase success of reaching promotion.^39^

### Funding, Resources, and Implementation

Of the 23 programs that provided information on funding, resources and implementation, many gave funding for early-career researchers through salary, stipends and scholarships, pilot research funding, travel/conference support, funds for publication fees, or equipment, supplies, and software.^36,42–44,47,53,55,57,63,69,71,72,74,79,85^ However, funding for mentors was mentioned in only four articles.^32,40,42,79^ Other program resources included workshops, clinician and facility visits, statistics and data support, and support for research presentations and project management.^51,69,70,79^ One of the Programs to Increase Diversity among Individuals Engaged in Health-Related Research (PRIDE) determined that leadership needed to provide theory-based research training, protected time, and a robust research infrastructure to URM faculty.^27^

DEI programs faced challenges in program implementation. Challenges included recruitment of specific URM groups,^68^ creating a streamlined application process,^59^ and ensuring that program participants have the requisite skills to succeed in the program.^84^ Duke University faced multiple hurdles in mounting its DEI program,^51^ so Duke pursued a multi-pronged approach including targeted outreach to investigators, partnerships with other institutional entities, structured assistance on grant applications, a KL2 program providing consistent mentorship, protected research time, and peer support.

### Community Engagement

Seven articles included a community engagement element in the DEI program. Community engagement initiatives helped to provide research staff jobs and training to diverse patient stakeholder data collectors,^61^ develop partnerships to collect data to address community health disparities,^25^ and train translational researchers in patient community outreach and engagement.^65^ The University of New Mexico’s Transdisciplinary Research, Equity and Engagement (TREE) Center faced obstacles including insufficient 1-year timeframe, lack of clear time commitment goals, scarce faculty mentors with limited community experience, lack of grant writing skills among mentees, and institutional barriers to multi-campus initiatives.^36^ Regardless, the TREE Center successfully funded ten projects led by a diverse cohort of investigators.

### DEI Program Practices

Practices common across many programs in this review included mentoring, research skills training, and social network facilitation.

### Mentoring

Mentoring was a core element of most programs (*n*=49).^24,27,28,33,36,38,40–44,47,48,50,53–57,59,61,64–67,69,70,73–78,80–82,84^ Mentoring took the form of peer-to-peer mentoring,^53,59,78,82^ group-mentoring,^27,54^ or a multiple mentoring approach where individuals were paired with a combination of mentors including research, career, institutional, and community mentors.^24,36,51^ Early-career researchers were often paired with senior investigators to develop leadership, research, and management skills. A few programs focused on providing training to mid- and senior career faculty on how to effectively mentor mentees from underrepresented groups.^32,52,74,77^

Despite the prominence of mentoring as a strategy, programs often struggled with identifying and engaging mentors.^42,47,69^ First, the dearth of URM senior scholars make them challenging to recruit to mentoring because they are often over-extended.^47^ Second, mentors who lacked salary coverage for their mentoring time were unable to provide adequate and sustained support to mentees.^27^ In an evaluation of the UCLA HIV/AIDS, Substance Abuse, and Trauma Training Program, participants wished for mentors to provide one-on-one time, share their own successful career paths, and provide preparation for career development awards, e.g., K01 grants.^63^

### Skills Training

Research skills training was seen as a positive step toward engaging people of diverse backgrounds in research, anticipating that skills training would contribute to their competitiveness in academic and research sectors. As such, skills training played a role in all 62 programs (Table 2). Didactic training was the most prominent format, used to introduce the terminology and rhetorical patterns found in NIH-style proposals or to integrate diverse views into the curriculum.^24,25,29,30,32,35,36,38,44,47–50,52,55,56,59,61,64–70,72,79,82,84^ Grant development training was another common offering,^29,35,41,45,48,52,53,58,61,65–67,71–74,76,81,84^ for example, through peer review of grant proposals or training on NIH grant procedures.^53^ Other forms of training involved clinical and methodological skills (e.g., building databases) or skills related to engaging patient communities in research. Some programs included bench research skills.^38,68^ The Healthy Brain Research Network contributed to development of communication and collaboration skills, often deemed “invisible” yet still crucial in research teamwork.^27,35^

The challenges in implementing skills building within programs included inadequate curricula, lack of qualified URM applicants, and limited grant writing skills.^36,61^ For the Strategic Empowerment Tailored for Health Equity Investigators grant writing program, one of the main challenges identified in the earliest cohorts was that applicants were not ready to develop a proposal that could be submitted within the duration of the program. Barriers to readiness included inadequate time, limited research support at their home institution, and shifting grant direction. To overcome the challenge of readiness, program leaders enhanced screening to identify participants who were closer to grant development.^91^ Other strategies to improve skills building included building a network among underrepresented colleagues to support each other in skill development,^41,91^ and tailoring training to the researchers’ career stage and prior experience.^81^

### Social Network Facilitation

Social networking was addressed in 11 articles. Networking programs funded attendance for early-career researchers at annual conferences or network meetings.^37,43,44,59,82^ One successful network facilitation was NIH’s National Institute of Diabetes and Digestive and Kidney Diseases (NIDDK) which launched a Network of Minority Health Research Investigators (NMRI) to connect URM researchers nationally and promote minority health research.^28^

In facing social network challenges, URM researchers who are junior faculty in teaching-intensive, minority-serving institutions often lack the time, training, and support to build professional networks. To meet this challenge, the Minorities Affairs Committee of the American Society for Cell Biology developed the VP Program to provide research training and access to professional networks.^34^ Another opportunity was the travel scholarship offered by the Society for Research on Nicotine and Tobacco (SRNT) Health Disparities Network for applicants from underrepresented groups. The scholarship funds attendance at the SRNT Annual Meeting and a 1-year membership.^37^

## DISCUSSION

This review of 62 studies documented program characteristics, success measures, infrastructure, and practices to improve DEI in the health and health services research workforce, while highlighting the challenges and opportunities sought over the past decade. Few of the 62 DEI programs in the review specifically focused on support for underrepresented group researchers based on their gender identity and sexual orientation,^74,75^ religion,^39^ or disability status.^37,39,41,46,50,56,67,68^ Additionally, only a handful of DEI programs focused on research staff.^25,39,49,54,60,61^ The primary metrics of program success included grants obtained and manuscripts published. The DEI programs reviewed mainly focused on career development through mentorship and skills training, or looking to increase leadership commitment and build sustained DEI funding and infrastructure. The expanding number of DEI programs attests to the importance of DEI advancement in health and health services research.

Only 40% programs tracked outcomes related to recruitment, promotion, and retention of underrepresented groups in the workforce pipeline. This may be partially attributed to program duration. Forty-seven percent of DEI programs were 1 year or less in length, limiting their ability to track long-term impacts on researcher careers. Our findings align with a review by Williams and colleagues on mentoring strategies to promote diversity, which found that less than a quarter of programs included outcomes related to career trajectory.^13^ There was also limited assessment of DEI program infrastructure (e.g., leadership support, sustained funding) and processes (e.g., mentorship quality, skills training) that enable success. A more innovative approach to program success, however, might involve examining how programs impact institutional culture and climate. As institutions consider implementing DEI metrics of their own, we recommend using a systematic process of identifying relevant and achievable metrics developed in partnership with individuals from underrepresented groups both at the institution and who are part of communities served. Some institutions have adopted DEI dashboards to track progress.^87,88^ We recommend that metrics are made transparent and regularly updated. Broad use and reporting of DEI metrics may lead to identification of institutions making progress in DEI and opportunities to share successful strategies.

A notable challenge is that all but one of the programs studied were funded by the US Government via NIH grants. This puts programs at risk because federal funding is often subject to budgetary cuts, delays, and non-renewals. For program sustainability, large research institutions should supplement or even match federal funds with state funds and internal funding from donors, alumni, non-profit collaborators, or other sources. Federal institutions must also increase their investment in initiatives which seek to increase DEI within the health and health service research workforce. Such strategies may include expansion of Career Development Awards^72^ and strengthening programs which provide pathways to staff or faculty for individuals from underrepresented groups. Efforts should also focus on rectifying racial and gender inequities in research grant receipt. Equity may be achieved through strategies such as prioritizing applications from investigators from underrepresented groups and minority serving institutions through higher payline thresholds.^89^ This strategy has been successfully used with granting priority to research proposals submitted by early stage investigators (ESI) within 10 years of completion of training. A study of the implementation of the ESI policy from 2011 to 2015 found that without the policy, 54% of ESIs would not have received grant funding.^90^

At a leadership and infrastructure level, research organizations can establish, fund, administer, measure, and report DEI progress at all phases of the workforce pathway with accountability at all levels (i.e., board and executive, school, department, and faculty/researcher). Leaders can center DEI in their institution mission statements to foster broad adoption, and advance DEI policies and practices through their operations and curricula. Cultural climate can be strengthened through inclusive workplace practices and access to physical, technological, and cultural resources that educate researchers for equitable decision-making and allow them participation in equitable work life.

Notable strengths of this review include the focus on multiple career roles and stages, attention to underrepresented groups across multiple identities, and the synthesis of program challenges and opportunities. Noting limitations of this review, scoping reviews aim to provide breadth of information on extant literature but do not assess its methodological quality.^91^ This review only included peer-reviewed empirical studies published in English in the USA over the past decade. Hence, unique and impactful programs and initiatives in other countries and those only reported in gray literature were not included. Future research should include systematic reviews and comparative effectiveness research to rigorously determine the effectiveness of varying DEI program types and strategies. Institutions which aim to initiate or expand DEI programs should also monitor whether such programs unintentionally lead to decreases in workforce DEI.

## CONCLUSIONS

Our review found that practices to improve DEI in the health and health services research workforce included career development through mentorship and skills training, investments in DEI infrastructure, and measurement efforts to document progress. While the literature offers some evidence of improved DEI toward building and sustaining a diverse workforce pathway, the institution-level, long-term work needed to expand the scope and sustained resources for DEI programs has just begun. Advancing longitudinal relevant and achievable program measurement for underrepresented groups at every career stage will contribute to greater DEI in the health and health services research workforce. This review provides an overview of strategies for consideration by institutions seeking to improve DEI within health and health services research.

## Supplementary Information

Below is the link to the electronic supplementary material.

Supplementary file1 (DOCX 76 KB)
