# Supplementary material for: Increasing Diversity, Equity, and Inclusion in the Health and Health Services Research Workforce: A Systematic Scoping Review
Source: J Gen Intern Med. 2024 Sep 25;40(7):1487–97. doi: 10.1007/s11606-024-09041-w (PMC12052632; doi:10.1007/s11606-024-09041-w)
Supplement: Supplementary file 1 — Supplementary file1 (DOCX 76 KB) [file 11606_2024_9041_MOESM1_ESM.docx]

**Appendix A – Search Terms and Strategy**

Dates of Study Publication: 01-01-2012 - 12-31-2022

Date of Search: 01-13-2023

Search Terms by Source:

**PubMed**

(((Research*[Title]) AND (Workforce*[Title/Abstract] OR "work force"[Title/Abstract] OR Employee*[Title/Abstract] OR Facult*[Title/Abstract] OR Staff*[Title/Abstract] OR "research assistant"[Title/Abstract] OR apprentice*[Title/Abstract])) AND (Divers*[Title/Abstract] OR Equit*[Title/Abstract] OR Inclus*[Title/Abstract] OR Represent*[Title/Abstract] OR Underrepresent*[Title/Abstract] OR Underserve*[Title/Abstract] OR Disparit*[Title/Abstract] OR Minorit*[Title/Abstract] OR Disab*[Title/Abstract] OR Race[Title/Abstract] OR Racial*[Title/Abstract] OR Ethnic*[Title/Abstract] OR Religio*[Title/Abstract] OR Socioecon*[Title/Abstract] OR Socio-econ*[Title/Abstract] OR Gender*[Title/Abstract] OR Impair*[Title/Abstract] OR Sex[Title/Abstract] OR "African American"[Title/Abstract] OR Black*[Title/Abstract] OR Hispan*[Title/Abstract] OR Latin*[Title/Abstract] OR "American Indian"[Title/Abstract] OR Alaska*[Title/Abstract] OR Native[Title/Abstract] OR Hawaii*[Title/Abstract] OR "Pacific Islander"[Title/Abstract] OR "Asian American"[Title/Abstract] OR Senior[Title/Abstract] OR Older[Title/Abstract]) AND ((2012:2022/12/31[pdat]) AND (english[Filter]))) NOT (undergraduate* OR "high school")

**1877 PubMed results**

**Embase**

research*:ti AND (workforce*:ab,ti OR 'apprentice* workforce*':ab,ti OR 'work force':ab,ti OR employee*:ab,ti OR facult*:ab,ti OR staff*:ab,ti OR 'research assistant':ab,ti OR apprentice*:ab,ti) AND (divers*:ab,ti OR equit*:ab,ti OR inclus*:ab,ti OR represent*:ab,ti OR underrepresent*:ab,ti OR underserve*:ab,ti OR disparit*:ab,ti OR minorit*:ab,ti OR disab*:ab,ti OR race:ab,ti OR racial*:ab,ti OR ethnic*:ab,ti OR religio*:ab,ti OR socioecon*:ab,ti OR 'socio econ*':ab,ti OR gender*:ab,ti OR impair*:ab,ti OR sex:ab,ti OR 'african american':ab,ti OR black*:ab,ti OR hispan*:ab,ti OR latin*:ab,ti OR 'american indian':ab,ti OR alaska*:ab,ti OR native:ab,ti OR hawaii*:ab,ti OR 'pacific islander':ab,ti OR 'asian american':ab,ti OR senior:ab,ti OR older:ab,ti) AND [2012-2022]/py AND ([article]/lim OR [article in press]/lim OR [review]/lim) AND [english]/lim NOT (undergraduate* OR 'high school')

**256 Unique Embase results** (not captured in PubMed search) out of **1584 Embase results**

**2133 results from PubMed and Embase** (The 2133 references were uploaded to Covidence which identified 136 more duplicates and 2 results as “not studies,” leaving 2131 articles.)

**2131 results from Database Search**

| **Appendix B – Overview of Diversity, Equity and Inclusion Programs in Health and Health Services Research** | | | | | | | | |
| --- | --- | --- | --- | --- | --- | --- | --- | --- |
| **#** | **Citation** | **Institution, Program, Funding and Scope, Analysis Frame, Program Length** | **Aims** | **Approaches to**  **Practice and Infrastructure** | **Underrep-resented Group(s)** | **Work-force Role(s)** | **Work-force**  **Goal(s)** | **Success Metric(s)** |
| 1 | Abebe  et al. 2019 | Institution: U. of Pittsburgh  Program: Career Education and Enhancement for Health Care Research Diversity (CEED)  Funding and Scope: NIH; Local  Analysis: 2007-2017  Program length: 1 year | Promote successful research careers for URM researchers; increase clinical and translational investigators in the biomedical research pipeline | Seminars,  Mentoring,  Research skills training,  Networking with Senior Faculty | Race/  ethnicity | Faculty | Hire | Papers,  Grants |
| 2 | Arrieta et al. 2018 | Institution: U. of South Alabama  Program: Bay Area Women Coalition, Inc.-USA Health Disparities Research Group (BAWC-HDRG) Collaboration  Funding and Scope: NIH; Local  Analysis: 2007–2010  Program length: 24 hours | Provide training and certification of a skills for employment in health research; enhance community understanding and engagement with health research | Research skills training,  Job offers | SES | Research staff | Hire | Research skills |
| 3 | Awad et al. 2022 | Institutions: Medical schools of U. of Puerto Rico; Howard U.; Tennessee State U.; Jackson State U.; U. of Hawaii; Morehouse U.  Program: NIH; National Institute on Minority Health and Health Disparities, Research Centers in Minority Institutions (RCMI)  Funding and Scope: NIH; National  Analysis: 2021-2022  Program length: N/A | Increase training capacity, career development, and institutional collaboration around data science techniques, resources, and tools | Computer science programming courses,  Seminars | Race/  ethnicity | Post-doctoral Fellows, Junior Faculty, Senior Faculty | -- | -- |
| 4 | Beech et al. 2018 | Institution: U. of Mississippi Medical Center  Program: Obesity Health Disparities, Programs to Increase Diversity among Individuals Engaged in Health-Related Research (PRIDE)  Funding and Scope: NIH; National  Analysis: N/A  Program length: N/A | Enhance research productivity to produce peer-reviewed publications, high-quality grant applications, general career development, and career navigation | Exposure to obesity research, One-on-one and group mentoring | Race/  ethnicity | Junior Faculty | -- | -- |
| 5 | Blanchard et al. 2019 | Institution: NIH; National Institute of Diabetes and Digestives and Kidney Diseases (NIDDK)  Program: Network of Minority Health Research  Investigators (NMRI)  Funding and Scope: NIH; National  Analysis: 2008-2018  Program length: N/A | Establish a network of minority health investigators in NIDDK related fields | Conferences,  Mentoring | Race/  ethnicity (Focus on AA, AI, AAPI, and Hispanic/  Latino) | Post-doctoral Fellows, Instruct-tors, and Junior Faculty | Promote | Grants |
| 6 | Boutjdir et al. 2019 | Institution: State U. of New-York, Downstate Medical-Center; Washington U. School of Medicine  Program: Programs to Increase Diversity Among Individuals Engaged in Health-Related Research (PRIDE)  Funding and Scope: NIH; National  Analysis: 2006-2018  Program length: 13-14 months | Provide mentoring, interdisciplinary training and research skills; Build ability to apply for independent funding; Develop successful academic careers and leadership positions | Research skills training, Mentoring, Grant writing | Race/  ethnicity, disability status | Junior Faculty | Promote | Papers,  Grants,  Research skills |
| 7 | Butler et al. 2017 | Institution: U. of Maryland at College Park; U. of Wisconsin-Madison  Program: Health Equity Leadership Institute (HELI)  Funding and Scope: NIH; National  Analysis timeframe: 2010-2016  Program length: 1 week | Increase the number of underrepresented scholars who can sustain their ongoing commitment to health equity research | Research skills training, Alumni network via blog, Facebook page, Emails | Race/ ethnicity | Post-doctoral Fellows, Lecturers Faculty | Promote  Retain | Grants |
| 8 | Butts et al. 2012a | Institution: Mt. Sinai  Program: Center for Multicultural and Community Affairs (CMCA)  Funding and Scope: NIH; Local  Analysis timeframe: 1998-2012  Program length: N/A | Expand and improve coordination of existing diversity efforts | N/A | Race/  ethnicity | Medical residents Faculty | Hire | Added  degrees |
| 8 | Butts et al. 2012b | Institution: Mt. Sinai  Program: Center for Multicultural and Community Affairs (CMCA) Faculty Scholars Program  Funding and Scope: NIH; Local  Analysis timeframe: 2001-2011  Program length: N/A | Equip URM faculty with the tools and information necessary for success in academic medicine | Research skills training,  Career development,  Funding for Masters programs,  Networking | Race/  ethnicity | Faculty | Hire | Added  degrees |
| 9 | Byars-Winston et al. 2018 | Institution: Four unnamed institutions  Program: Culturally Aware Mentoring  Funding and Scope: NIH; National  Analysis: 2016  Program length: 6 hours | Support research mentors’ confidence in mentoring historically underrepresented groups | Research training | Race/  ethnicity | Faculty | -- | Inclusive climate |
| 10 | Byington et al. 2017 | Institution: U. of Utah Center for Clinical and Translational Science (CCTS)  Program: Clinical and Translational Scholar program  Funding and Scope: NIH; Local  Analysis: 2008-2016  Program length: 2 years | Promote workforce development for translational research | Mentoring,  Research support services,  Networking | Gender, race/  ethnicity,  rurality, low-SES | Faculty | Retain | Grants,  Added degrees |
| 11 | Campbell et al. 2013 | Institution: Minorities Affairs Committee of the American Society for Cell Biology (ASCB)  Program: Visiting Professor  Funding and Scope: NIH; National  Analysis: 1997-2011  Program length: 8-10 weeks | Strengthen research infrastructure and teaching practices at minority serving institutions (MSIs) | Career development,  Lab internship,  Teaching support,  Networking | Race/  ethnicity, gender | Faculty | Promote | Papers,  Grants |
| 12 | Croff et al. 2020 | Institution: US Centers for Disease Control and Prevention  Program: Healthy Brain Research Network (HBRN) Scholars Program  Funding and Scope: NIH; Local  Analysis: 2015-2018  Program length: Varies by institution | Engage diverse scholars in mentored, interdisciplinary research and national collaboration | Research skills training, Research project,  Grant writing, Community work,  Networking | Race/  ethnicity, gender | Post-doctoral Fellows | Hire | Papers,  Grants |
| 13 | Cruz et al. 2020 | Institution: Transdisciplinary Research, Equity and Engagement Center (TREE) for Advancing Behavioral Health  Program: Pilot Program  Funding and Scope: NIH; Local  Analysis: 3 years  Program length: 1 year | Provide skills, community partnerships, and preliminary data needed to address behavioral health disparities | Mentoring,  Research skills training,  Seminars,  Funding | Race/  ethnicity, gender | Faculty | Hire | Papers,  Grants |
| 14 | Davis et al. 2020 | Institution: Society for Research on Nicotine and Tobacco (SRNT) Health Disparities Network  Program: The SRNT Health Disparities Network  Funding and Scope: NIH; National  Analysis: 2007-2014  Program length: 1 year | Provide scholarships to early career investigators who conduct research that addresses tobacco-related health disparities that affect marginalized and minority populations | Conferences,  One-year SRNT membership | Race/  ethnicity, disability, sexual orienta-tion, SES,  gender identity | Junior Faculty | -- | Papers |
| 15 | Diallo et al. 2021 | Institution: The PRIDE Cardiovascular Disease Summer Institute at State U. of New York  Program: The PRIDE CVS  Funding and Scope: NIH; National  Analysis: June 14-26, 2020  Program length: 2 weeks | Support URM faculty in retention and career development during the pandemic | Research skills training,  Mentoring,  Wellness sessions | Race/  ethnicity | Junior faculty | -- | -- |
| 16 | Enders et al. 2021 | Institution: Mayo Clinic Department of Health Sciences Research  Program: Diversity, Equity, and Inclusion (DEI) Plan  Funding and Scope: NIH; Local  Analysis: July 2020  Program length: N/A | Increase overall and subgroups’ sense of belonging and Increase overall diversity | DEI Training for all HSR leaders and employees,  Record DEI feedback for leaders, DEI in performance reviews | Race/  ethnicity, gender, disability, national-ity, sexual orienta-tion, religion | Faculty, ResearchStaff | -- | -- |
| 17 | Estape et al. 2018 | Institution: U. of Puerto Rico-Medical Sciences Campus; Morehouse School of Medicine  Program: NIH Clinical Research Education and Career Development (CRECD) R25 award program  Funding and Scope: NIH; National  Analysis: 2003-2017  Program length: 3-5 years | Increase diversity in gender, race/ethnicity, and disciplines by supporting scholars in Clinical and Translational Research or Clinical Research; expand mentor and scholar pool through multiple channel recruiting | Research training (in-person/online)  Mentoring,  Salary funding for mentors,  Funding for scholars,  Eval/Progress reports | Race/ ethnicity, gender | Post-doctoral Fellows | Hire  Promote  Retain | Grants |
| 18 | Fabris et al. 2016 | Institution: State U. of New-York Downstate-Medical Center at Brooklyn  Program: Summer Institute Program to Increase Diversity in Health-Related Research (SIPID)/ Program to Increase Diversity Among Individuals Engaged in Health-Related Research (PRIDE)  Funding and Scope: NIH; National  Analysis: 2006-2015  Program length: 2 summers (3 weeks) | Train multidisciplinary scientists and clinicians from racial racial/ethnic groups and/or groups with disabilities that are underrepresented in biomedical and behavioral sciences | Research skills training, Mentoring, Grant writing, Career development | Race/ethnicity, disability | Faculty | Promote | Papers,  Grants |
| 19 | Fernandez et al. 2016 | Institution: NIH  Program: HIV Prevention Trials Network (HPTN)  Funding and Scope: NIH; National  Analysis: 2010-2015.  Program length: 18 months | Increase the number of U.S. underrepresented scholars who are successful HIV prevention researchers | Manuscript development, Mentoring, Scholar and mentor funds | N/A | Faculty | -- | Papers,  Grants |
| 20 | Flores et al. 2021 | Institution: NIH; National Institute  of Diabetes and Digestive and Kidney Disorders (NIDDK)  Program: Research in Academic Pediatrics Initiative on Diversity (RAPID)  Funding and Scope: NIH; National  Analysis: 2012-2017  Program length: 1 year | Recruit, retain, and professionally advance diverse early-career faculty in general pediatrics who are pursuing research careers | Research grants, Mentoring, Networking, Conferences | Race/ethnicity | Faculty | -- | Papers,  Mentor-ship quality |
| 21 | Goldstein et al. 2014 | Institution: American Psychiatric Association  Program: Program for Minority Research Training Program in Psychiatry (PMRTP)  Funding and Scope: NIH; National  Analysis timeframe: 1989-2010  Program length: N/A | Support psychiatric investigators from URM groups in the development and maintenance of research careers; identify factors that influence successful research career development | Stipends for trainees to be placed at host sites, Research skills training, Conferences, Mentoring | Race/  ethnicity | Post-doctoral Fellows | Hire | Papers,  Grants |
| 22 | Grant et al. 2022 | Institution: Two tribal colleges and one public, non-tribal, PhD-granting institution in the Northern Great Plains  Program: Native American Faculty in Science, Technology, Engineering, and Mathematics (NAF-STEM)  Funding and Scope: Regional  Analysis timeframe: 2018-2021  Program length: 1 year | Apply the six R’s framework for Indigenous research (Respect, Relationship,  Representation, Relevance, Responsibility, Reciprocity) to career development in research | Grant writing, Collaborative writing and support group, Research publication program | Race/  ethnicity | Faculty | -- | Papers,  Grants |
| 23 | Gwath-mey et al. 2016 | Institution: Wake Forest U. - School of Medicine and School of Arts and Sciences  Program: Post-Doctoral Research, Instruction, and Mentoring Experience (PRIME)  Funding and Scope: NIH; Local  Analysis timeframe: 2013-2016  Program length: 3 years | Increase the diversity of academic researchers in the medical/allied health professions; train scholars to use innovative approaches in teaching students from underrepresented backgrounds | Research skills training, Mentoring, Pedagogical training | Race/  ethnicity, disability, SES | Post-doctoral Fellows | Hire | -- |
| 24 | Harawa et al. 2017 | Institution: NIH; National Institute on Aging (NIA)  Program: The Resource Centers for Minority Aging Research (RCMAR) program  Funding and Scope: NIH; National  Analysis: 1997-2015  Program length: 2 years | Across seven centers, increase the number of qualified researchers focused on the health of aging racial and ethnic minorities; enhance scientific workforce diversity through mentorship and career development | Mentoring,  Research skills training,  Content training on aging and health disparities | Race/  ethnicity | Faculty | Retain | Papers,  Grants |
| 25 | Ingram et al. 2021 | Institution: Multiple universities in South Carolina  Program: Carolina Center on Alzheimer's Disease and Minority Research  Funding and Scope: Statewide  Analysis: 2018-2020  Program length: 1 year | Fund and mentor pilot projects for URM scientists conducting research on Alzheimer’s disease and related dementias; offer research education in health disparities and minority aging research | Pilot project, Mentoring, Research skills  training,  Content training on aging and health disparities | Race/  ethnicity | Faculty | -- | Grant |
| 26 | Javier et al. 2022 | Institution: NIH; National Research Mentoring Network (NRMN)  Program: Unconscious Bias Course  Funding and Scope: NIH; National  Analysis: 2020-2021  Program length: 1 hour, 40 mins | Educate on individual bias and existing disparities in healthcare; offer course on: unconscious bias, micro-aggressions, solutions toolkit, self-awareness, bias and disparities in healthcare | Content training (online) on unconscious bias and healthcare disparities | Race/  ethnicity | Faculty, ResearchStaff | -- | -- |
| 27 | Jean-Louis et al. 2016 | Institution: NYU Langone Medical Center  Program: PRIDE Institute in Behavioral Medicine and Sleep Disorders Research  Funding and Scope: NIH; National, externally funded  Analysis: 2010-2014  Program length: ~1 year | Provide mentored research training to junior URM faculty | Research skills training, Mentorship, Grant development | Race/  ethnicity, disability | Faculty | -- | Papers,  Grants,  Research skills |
| 28 | Johnson et al. 2021 | Institution: Duke U.  Program: NIH KL2 Research Career Development Award  Funding and Scope: NIH; Local  Analysis: 2013-2019  Program length: 3 years | Increase the number of investigators from underrepresented racial and ethnic groups | Mentoring, Research skills training | Race/  ethnicity | Junior Faculty | Retain | Grants |
| 29 | Johnson et al. 2015 | Institution: U. of California, San Francisco  Program: Mentoring the Mentors workshop  Funding and Scope: NIH; National  Analysis: 2013 before the workshop and two weeks after  Program length: 2 days | Enhance mentoring techniques and topics related to diversity | Mentorship training | Race/  ethnicity | Senior Faculty | -- | Mentor- ship quality |
| 30 | Jones et al. 2017a | Institution: NIH/ NIH; National Research Mentoring Network (NRMN); U. of Minnesota  Program: The Proposal Preparation Program (NRMN-P  Funding and Scope: NIH; National  Program Length: 4-5 months | Support the professional advancement of Post-doctoral Fellows and Junior Faculty from diverse populations by offering training and coaching in grant-writing and career development | Research skills training by coach, Peer and mentor grant proposal review, Mock NIH study section | Race/  ethnicity | Faculty | -- | ­­-- |
| 30 | Jones et al. 2017b | Institution: NIH/NRMN Northwestern U. Feinberg School of Medicine  Program: Northwestern U. Grant writers Coaching Groups (NU)  Funding and Scope: NIH; National  Program Length: 3-4 months | Offer trainees substantial practice in using the terminology and rhetorical patterns typically found in NIH-style proposals to convey key messages | Ibid. | Race/  ethnicity | Post-doctoral Fellows, Junior Faculty | -- | -- |
| 30 | Jones et al. 2017c | Institution: NIH/NRMN; U. of Colorado Anschutz Medical Campus; Washington State U.  Program: Grant writing Uncovered: Maximizing Strategies, Help, Opportunities, Experiences (GUMSHOE)  Funding and Scope: NIH; National  Analysis Frame: N/A  Program Length: 6 months | Provide support for grant development related to health disparities | Ibid. plus Feedback from NIH Grant Program Officials (GPO), Mentee collaboration | Race/  ethnicity | Faculty | -- | -- |
| 30 | Jones et al. 2017d | Institution: NIH/NRMN; U. of North Texas Health Science Center -- Texas Center for Health Disparities  Program: Steps Towards Academic Research (NRMN STAR)  Funding and Scope: NIH; National  Analysis: N/A  Program length: 1 year | Foster the relationships needed for effective health disparities research and to promote education and training for academic institutions | Research skills training, Grant proposal development, Mentoring,  Peer-to-peer working group | Race/  ethnicity | Post-Doctoral Fellows, Junior Faculty | -- | -- |
| 31 | Kolb et al. 2022 | Institution: U. of Florida Clinical and Translational Science Institute; Ohio State U. Center for Clinical Translational Science  Program: DEI co-mentoring circles  Funding and Scope: NIH; National  Analysis: N/A  Program length: 6 months | Create a model that stimulates reflection as well as sharing and solution- finding for diversity, equity, and inclusion issues of the workforce in clinical research management work | Group mentoring sessions, Black Voices in Research videos | Race/  ethnicity | Staff | -- | Inclusive climate |
| 32 | Kuo et al. 2017 | Institution: UCLA Center for Population Health and Health Disparities  Program: UCLA Center for Population Health and Health Disparities (CPHHD) Training and Career Development program  Funding and Scope: NIH; Local/regional Program length: N/A | Provide to Fellows: Stipends, Access to project data, Statistical support for publications, Mentoring, Teaching and mentoring opportunities. Provide to Junior Faculty: Grant writing, Mentorship for publications, Teaching opportunities | Funding, Mentoring, Statistical support, Grant writing training, Teaching and mentoring opportunities | Race/  ethnicity (Latino) | Post-doctoral Fellows, Junior Faculty | -- | Papers,  Grants |
| 33 | Leon Guerrero et al. 2017 | Institution: NIH Diversity Program Consortium (DPC)  Program: NIH; National Research Mentoring Network (NRMN)  Funding and Scope: NIH; National  Analysis timeframe: N/A  Program length: N/A | Increase the representation and success of underrepresented groups in biomedical research by enhancing the training and career development | Mentoring, Mentorship training, Career and research resources | Race/  ethnicity, disability, SES | Post-doctoral Fellows, Faculty | -- | -- |
| 34 | Leon Guerrero et al. 2020 | Institution: U54 Partnership between the U. of Guam and the U. of Hawai'i Cancer Center  Program: The UOG/UHCC Partnership Funding and Scope: Regional Program length: 2 years | Develop researchers from underrepresented minorities while increasing number of Indigenous Pacific peoples with successful careers addressing cancer prevention/control in island communities in the Pacific | Salary funding,  Research skills training, Mentorship, Career development, Conferences | Race/  ethnicity (Indigen-ous Pacific peoples) | Junior Faculty | -- | Papers,  Grants |
| 35 | Levites Strekalova et al. 2021 | Institution: N/A  Program: Black Voices in Research curriculum  Funding and Scope: NIH; National Analysis: N/A  Program length: N/A | Provide instructional materials to: facilitate DEI dialog in biomedical research, enhance critical thinking, integrate diverse visions and worldviews, and promote action | Content to support DEI understanding and action in biomedical research | Race/  ethnicity | Faculty, Research Staff | -- | Inclusive climate |
| 36 | Limaye et al. 2019 | Institution: District of Columbia Centers for AIDS Research (CFAR), Johns Hopkins U. CFAR, and U. of Pennsylvania CFAR  Program: Mid-Atlantic CFAR Consortium (MACC) Scholars Program  Funding and Scope: Regional (Mid-Atlantic)  Analysis: N/A  Program length: N/A | Support underrepresented scientists in research to address the HIV epidemic in the Mid-Atlantic region; prepare Junior Faculty at CFAR institutions or junior scientists to write competitive CFAR pilot proposals, K- or R-series, or join other Mentors | Salary funding,  Mentorship, Grant writing, Peer support on grants and publications, Pilot project funding, Conferences, Publication fees, Trips to CFARS centers | Race/  ethnicity | Junior Faculty | -- | Papers,  Grants |
| 37 | Lininger et al. 2022 | Institution: Southwest Health Equity Research Collaborative of the Research Centers for Minority Institutions  Program: Technical Assistance Group-Service Center  Funding and Scope: NIH; National  Analysis: N/A  Program length: N/A | Build a service center with faculty and staff who are expert in research design and methodology as a resource for minority health research institutions | Research skills training,  Data management,  REDCap assistance | Race/  ethnicity | Faculty,  Post-doctoral Fellows, Research Staff | -- | Grants |
| 38 | McGrew et al. 2020 | Institution: U. of New Mexico; One Hope Centro de Vida Health Center  Program: N/A  Funding and Scope: NIH; Local  Analysis: 2016-2019  Program length: 70 hours | Train patient stakeholder data collectors from low-income Latino households to improve research equity; provide career opportunities to community members | Community partner-led hiring and training of patient stakeholder data collectors | Race/  ethnicity (Hispanic/Latino), Language (Spanish),  SES | Research  Staff | Hire | -- |
| 39 | Meadows et al. 2022 | Institution: Meharry Medical College, Tennessee State U.; Vanderbilt Ingram Cancer Center  Program: Meharry-Vanderbilt-TSU Cancer Partnership’s NIH; National Cancer Institute Comprehensive Partnerships in Advancing Cancer Health Equity (CPACHE)  Funding and Scope: NIH; National  Analysis: 2011-2021  Program Length: N/A | Increase the number of underrepresented Post-doctoral Fellows and Medical Residents; increase cancer research and education capacity of institutions serving underserved health disparity populations | Structured research experiences | Race/  ethnicity, Gender | Post-doctoral Fellows, Medical Resident | Hire, Retain | -- |
| 40 | Milburn et al. 2019 | Institution: U. of California, Los Angeles  Program: HIV/AIDS, Substance Abuse, and Trauma Training Program (HA-STTP) Funding and Scope: NIH; National  Analysis: 2014-2018  Program length: 2 years | Increase the number of early-career behavioral scientists conducting research on HIV/AIDS, substance abuse, and traumatic stress; ensure continued career and research success | Research skills training, Mentorship, Grant proposal development, Funding for pilot studies, resources and conferences | Race/  ethnicity | Junior Faculty, Post-doctoral Fellows | -- | Papers,  Grants |
| 41 | Ofili et al. 2021 | Institution: NIH  Program: Research Centers in Minority Institutions Translational Research Network (RCMI RTRN)  Funding and Scope: NIH; National  Analysis: 2000–2015  Program length: N/A | Unite the member minority-serving universities, colleges of pharmacy and veterinary medicine and medical schools conducing translational research to address health disparities in underserved communities; develop governance and policy administration, cross-institutional collaboration, funding, study and data management resources | Steering committee to develop policy, Research clusters for collaboration across institutions,  Regulatory compliance training, Pilot funding, Study and data management,  Biostatistics support | Race/  ethnicity | N/A | -- | Papers |
| 42 | Oldham et al. 2020 | Institution: Duke Cancer Institute and North Carolina Central U.  Program: Cancer Research Education Program (C-REP)  Funding and Scope: Regional  Analysis: 2018-2019  Program length: 2 years | Recruit and train translational scientists so they may increase URM participants in clinical trials; strengthen community outreach/engagement as a focus in translational research and train researchers accordingly | Research skills  training, Mentoring, Facility tours, Shadowing clinicians, Community outreach, Networking | Race/  ethnicity | Post-doctoral Fellows | Hire | Papers,  Grants,  Research skills |
| 43 | Pace et al. 2017 | Institution: Augusta U.  Program: Functional and Translational Genomics of Blood Disorders Program to Increase Diversity Among Individuals Engaged in Health-Related Research (FTG-PRIDE)  Funding and Scope: NIH; National  Analysis: 2006-2016 Program length: 2 years | Train Junior Faculty in grant writing to achieve extramural funding and in research-related technical skills to investigate functional and translational genomics of blood disorders | Mentoring, Research and lab skills training, Networking, Funding and Grant development, Conferences | Race/  ethnicity | Junior Faculty | Promote | Grants,  Research skills  Teaching positions |
| 44 | Rice et al. 2017 | Institution: NIH; National Heart Lung and Blood Institute (NHLBI)  Program: Summer Institute Programs to Increase Diversity (SIPID) and Programs to Increase Diversity Among Individuals Engaged in Health-related Research (PRIDE) Funding and Scope: NIH; National  Analysis: 2007-2016  Program length: 2 summers (2-3 weeks) | Train Junior Faculty from underrepresented researcher groups to better write scientific articles, prepare competitive grant applications, and in the long term, gain research independence | Research/lab skills training, Grant writing, Networking, Mentoring, Mock grant review,  PRIDE only: Conferences | Race/  ethnicity, disability, SES | Junior Faculty | Promote | Papers,  Grants,  Research skills |
| 45 | Rice et al. 2014 | Institution: NIH; National Heart Lung and Blood Institute (NHLBI)  Program: Summer Institute Programs to Increase Diversity (SIPID)  Funding and Scope: NIH; National  Analysis: 2007-2010  Program length: 2 summers (2-3 weeks) | Enable junior faculty to develop their research skills, subject knowledge and enhance their career development in heart, lung and blood research. | Research skills  training, Grant development, Mock study section, Mentoring, Development of 5-year plans | Race/  ethnicity, disability | Junior Faculty | -- | Papers,  Grants |
| 46 | Rubio et al. 2019 | Institution: Medical schools of U. of Pittsburgh; Charles R. Drew U.; Howard U.; Morehouse U.; U. of Hawaii Manoa; U. of Puerto Rico  Program: Leading Emerging and Diverse Scientists to Success (LEADS)  Funding and Scope: NIH; National  Analysis: N/A  Program length: 1 year | Help early-career scientists at Minority Serving Institutions launch successful biomedical research careers, thus expanding research capacity at their home institutions. | Research skills training | Race/  ethnicity | Post-doctoral Fellows, Junior Faculty | -- | -- |
| 47 | Salama et al. 2021 | Institution: Duke U.  Program: Duke Radiation Oncology Research Scholar (RORS) Program  Funding and Scope: NIH; Local (NIH; National Cancer Institute)  Analysis: N/A  Program length: 2 years | Eliminate barriers preventing radiation oncology trainees from becoming independent physician scientists | Mentoring, Training, Funding for salary and research | Race/  ethnicity, gender | Junior Faculty | -- | -- |
| 48 | Sensoy Bahar et al. 2021 | Institution: Washington U., St. Louis, MO  Program: Training LEADers to Accelerate Global Mental Health Disparities Research (LEAD) program  Funding and Scope: NIH; National  Analysis: June 2020-September 2021  Program length: 12 weeks | Provide evidence-based, culturally and contextually congruent training by conducting field-based research in Sub-Saharan Africa to address global mental health disparities | Research skills training,  Field-based research, Mentorship, Networking, Conferences | Race/  ethnicity | Junior Faculty | -- | Papers,  Grants |
| 49 | Singh et al. 2022 | Institution/Program: NIH; National Institute of Diabetes and Digestive and Kidney Diseases (NIDDK)  Funding and Scope: NIH; National  Analysis: N/A  Program length: N/A | NIDDK emphasizes diversity and inclusion in research of Diabetes and Digestive and Kidney Diseases. It supports the Network of Minority Health Research Investigators (NMRI) | Salary funding,  Grant funding supplements,  Mentoring,  Research skills training,  Career development,  Conferences | Race/  ethnicity, gender | Post-doctoral Fellows, Junior Faculty, Senior Faculty | -- | -- |
| 50 | Smith et al. 2017 | Institution: Montana State U.  Program: Grant-writing bootcamp for women in STEM  Funding and Scope: NIH; Local  Analysis: 18 months  Program length: 6 months | Support the women faculty participants’ feelings of relatedness, autonomy, and competence, to improve research submission rates and funding success | Grant-writing, Training | Gender | Faculty | -- | Grants |
| 51 | Sorkness et al. 2017 | Institution: NIH  Program: NIH; National Research Mentoring Network (NRMN)  Funding and Scope: NIH; National  Analysis Timeframe: N/A  Program Length: N/A | Establish a national training hub to support mentorship and career development in the biomedical, behavioral, clinical, and social sciences, emphasizing diversity, inclusivity, and culture in mentoring relationships and the research workforce | Mentoring, Grant development support, Coaching | Race/  ethnicity, gender, sexual orienta-tion,  SES | Post-doctoral Fellows, Junior Faculty, Senior Faculty, Research Staff, Adminis-trators | -- | -- |
| 52 | Sutton et al. 2013 | Institution: Centers for Disease Prevention and Control (CDC)  Program: Minority HIV/AIDS Research Initiative (MARI)  Funding and Scope: CDC; National  Analysis: 2003-2013  Program length: 3 years | Provide competitively awarded, mentored grants to early career, URM scientists performing HIV prevention research in highly affected racial/ethnic and sexual minority communities | Grant funding, Scientific Training, Mentoring,  Technical Assistance, Networking | Race/  ethnicity, gender, sexual orienta-tion | Junior Faculty | Retain | Papers,  Grants |
| 53 | Thorpe et al. 2020 | Institution: The NIH; National Research Mentoring Network (NRMN)  Program: Steps Toward Academic Research (NRMN STAR) Fellowship Program  Funding and Scope: NIH; National  Analysis: 18 months  Program Length: 1 year | Improve self-efficacy in NIH-level grant writing among underrepresented minorities in the biomedical and behavioral science research workforces | Research skills training, Coaching, Grant development, Working groups | Race/  ethnicity | MD/PhD Post-doctoral Fellows, Junior Faculty | -- | Research skills |
| 54 | Trejo et al. 2022 | Institution: School of Medicine, U. of California, San Diego, CA  Program: Faculty mentor training program (FMTP) across UC San Diego Health Sciences  Funding and Scope: NIH; Local  Analysis: 2017-2020  Program Length: 1 year | Improve mentoring quality, faculty satisfaction, and inclusive culture for health sciences faculty at all levels | Institutional support, Mentorship training at the individual, departmental division levels | Race/  ethnicity | Junior Faculty, Senior Faculty | -- | Inclusive climate,  Mentor-ship quality |
| 55 | Varkey et al. 2012 | Institution: Department of Medicine at Mayo Clinic  Program: Facilitated peer mentoring of women physicians  Funding and Scope: NIH; Local  Analysis: N/A  Program Length: 1 year | Improve quality and quantity of publications by women physicians in peer-reviewed journals | Mentoring by Senior Faculty and Peers, Manuscript development, Research skills training | Gender | Junior Faculty | -- | Papers,  Research skills |
| 56 | Velasquez et al. 2019 | Institution: NIH; National Institute of Allergy and Infectious  Diseases, AIDS Clinical Trials Group  Program: The Minority HIV Investigator Mentoring Program (MHIMP)  Funding and Scope: NIH; National  Analysis: 1996-2017  Program Length: 1 year | Help minority junior investigators jumpstart their careers as HIV investigators | Mentoring, Grant development, Salary funding for mentors and scholars, Networking, Conferences | Race/  ethnicity | Junior Faculty | Promote | Papers,  Grants |
| 57 | Vermund et al. 2018 | Institution: HIV Prevention Trials Network (HPTN)  Program: HIV Prevention Trials Network  (HPTN) Scholars Program  Funding and Scope: International  Analysis: 2016-2018  Program Length: 12-18 months | Assist the scholar in navigating the complex systems of network research and to promote self-efficacy that will enable scholars to move toward longer term success and retention in HIV prevention research | Mentoring, Manuscript development, Community engagement, HPTN scientific committees, Research skills training, Coaching | Race/  ethnicity, sexual orienta-tion | Junior Faculty (MD and Post-doctoral Fellows) | Retain | Papers,  Grants |
| 58 | Vishwa-natha et al. 2018 | Institution:  Texas Center for Health Dis­parities; NIH; National Research  Program: Steps Toward Academic Research (NRMN STAR) Fellowship Program  Funding and Scope: NIH; National  Analysis: 2008-2016  Program Length: 1 year | Pursue health disparity research; provide faculty and community partners face-to-face and online curriculum to acquire fundamental knowledge and skills in biomedical and behavioral health disparity research, grantsmanship, and career development | Pilot grant,  Research skills training,  Conferences | Race/  ethnicity | Junior Faculty and Commu-nity stake-hold­ers | Promote | Grants |
| 59 | Walsh et al. 2021 | Institution: Cornell U., Weill  Cornell Medicine  Program: Female Global Scholars Program  Funding and Scope: NIH; Local  Analysis: April 2018-March 2020  Program Length: 2 years | Promote the advancement of female global health research professionals and prepare them for leadership positions in this field | Research skills training, Peer mentoring, Manuscript development, Conferences | Gender | Junior Faculty | Promote | Papers |
| 60 | Weber-Main et al. 2022 | Institution: U. of Utah School of Medicine  Program: NIH Diversity Program Consortium  Funding and Scope: NIH; National  Analysis: 2 years after each cohort’s cycle  Program length: 18 months | Enhance the participation and persistence of individuals from underrepresented backgrounds in biomedical research careers | Virtual coaching,  In-person Research skills training | Race/  ethnicity | Junior Faculty | -- | Grants |
| 61 | Weber-Main et al. 2020a | Institution: U. of Minnesota; NIH; National Research Mentoring Network (NRMN)  Program: NRMN Proposal Preparation Program (P  Funding and Scope: NIH; National  Analysis: 2015-2019  Program length: 5 months | See Jones et al. 2017a | | | | -- | Grants |
| 61 | Weber-Main et al. 2020b | Institution: U. of North Texas Health Sciences Center  Program: NRMN Steps Towards Academic Research (STAR)  Funding and Scope: NIH; National  Analysis: 2015-2019  Program length: 10 months | See Jones et al. 2017d | | | | -- | Grants |
| 61 | Weber-Main et al. 2020c | Institution: Morehouse School of Medicine  Program: NRMN Strategic Empowerment Tailored for Health Equity Investigators (SETH)  Funding and Scope: NIH; National  Analysis: 2015-2019  Program length: 6 months | Provide diverse cohorts of Junior Faculty with sustained and intensive coaching in grant proposal development | Coaching /mentoring, Grant development, Virtual community, Resource sharing | Race/  ethnicity, gender | Junior Faculty | -- | Grants |
| 61 | Weber-Main et al. 2020d | Institution: U. of Chicago  Program: NRMN Committee on Institutional Cooperation Academic Network (CAN)-  Funding and Scope: NIH; National  Analysis: 2015-2019  Program length: 6 months | Provide diverse cohorts of Junior Faculty with sustained and intensive coaching in grant proposal development | Research skills training, Coaching /mentoring, Grant Development | Race/  ethnicity, gender | Junior Faculty | -- | Grants |
| 61 | Weber-Main et al. 2020e | Institution: U. of Colorado  Program: NRMN Maximizing Strategies, Help, Opportunities, and  Experiences (GUMSHOE)-  Funding and Scope: NIH; National  Analysis: 2015-2019  Program length: 6 months | See Jones et al. 2017c | | | | -- | Grants |
| 61 | Weber-Main et al. 2020f | Institution: Northwestern U.  Program: NRMN Northwestern U. Grant Writers Coaching Groups (NU)  Funding and Scope: NIH; National  Analysis: 2015-2019  Program length: 4-6 months | See Jones et al. 2017b | | | | -- | Grants |
| 62 | Williams et al. 2021 | Institution: Center for Clinical and Translational Science (CCTS)  Program: The Disparities Researchers Equalizing Access for Minorities (DREAM) program  Funding and Scope: NIH; National  Analysis: 2018-2020  Program length: 2 years | To promote the career development of multidisciplinary Pre-Docs, Post-doctoral Fellows and Junior Faculty who are interested in conducting health equity research among special populations | Research skills training, Writing retreat, Mentoring, Pilot funding, Conferences | Race/  ethnicity | Post-doctoral Fellows, Junior Faculty | -- | Papers,  Grants |

| **APPENDIX C– DEI Program Challenges and Opportunities** | | |
| --- | --- | --- |
| **#** | **Citation** | **Program Challenges and Opportunities** |
|  |  | **DEI Program Infrastructure** |
|  |  | **(1) Leadership and cultural climate – Challenges** |
| 5 | Blanchard et al, 2019 | For underrepresented minority investigators have difficulty in becoming independent researchers due to institutional requirements for clinical service, committee work, and ineffective time management. When funded, URM researchers may encounter other difficulties, including lack of skills to successfully recruit grant coordinators, negotiate dedicated time and space, manage a clinical trial, oversee a budget and payment, direct a team, and recruit and sustain appropriate sample size and statistical adequacy. |
| 8 | Butts et al, 2012 | A Faculty Diversity Climate Survey at the Mount Sinai School of Medicine (MSSM) found that the opinions of the ‘‘majority’’ population on the school’s diversity success were more positive than the opinions of those from underrepresented groups (e.g., respondents who self-identify as racial/ethnic minorities or women) on all attitude questions. |
|  |  | **(1) Leadership and cultural climate - Opportunities** |
| 7 | Butler et al, 2017 | University of Maryland at College Park and the University of Wisconsin-Madison established the Health Equity Leadership Institute (HELI) which offers a 1-week, evidence-based program for leaders to develop a diverse workforce for health equity research. |
| 9 | Byars-Winston et al, 2018 | Evidence supports the beneficial effects of cultural competence training on the attitudes and skills of health professionals. Research mentors who participated in cultural awareness training reported significant skill gains not only in their intentionality to address race/ethnicity but increased openness to broach racial, ethnic, and cultural topics in their research mentoring relationships and willingness to go outside of their comfort zone. |
| 16 | Enders et al, 2021 | Mayo Clinic leaders receive DEI training to address issues that arise from the hierarchical nature of leadership, introducing strategies to prevent problematic behaviors (e.g., bullying). Then every two years, the Leadership 180 review provides employee feedback to leaders with observations on DEI strengths and areas for improvements. DEI culture at Mayo Clinic was promoted through “inclusion nudges” built into leaders’ annual performance reviews. |
| 27 | Jean-Louis et al, 2016 | A scholar felt that the nature of the institute and the support given motivated her to want to do more than treating patients. The Institute was referred to as a “safe place to learn and make mistakes.” The PRIDE staff created an atmosphere of acceptance, but the mentees themselves added to the safe space. |
|  |  | **(2) Funding, resources and implementation - Challenges** |
|  |  | **Funding challenges** |
| 13 | Cruz et al, 2020 | There was a delay in funding due to extended NIH review time. |
| 19 | Fernandez et al, 2016 | Scholars and mentors are not compensated for their time. Program was discontinued. |
| 24 | Harawa et al, 2017 | - Declining NIH funding rates.  - Limited resources to compensate mentors might limit number of mentors available and the amount of time each can dedicate to mentees; limited availability of faculty positions; many mentors are not from URG. |
| 25 | Ingram et al, 2021 | Limiting budget support to graduate students is unintentionally punitive to post-doc applicants from schools that may not have a significant pool of graduate students matriculating on their campuses. |
| 28 | Johnson et al, 2021 | Variation in available resources and support limit success. |
| 36 | Limaye et al, 2019 | Insufficient funding was a barrier ($15,000 per scholar). |
| 47 | Salama et al, 2021 | Lack of funding, and inadequate institutional support. |
| 61 | Weber-Main et al, 2020 | Less grant support may be optimal only when individuals have strong scientific preparation or support systems in their home institutions. |
|  |  | **Resource challenges** |
| 3 | Awad et al, 2022 | - Lack of resources to support programming with representative datasets  - Difficulty finding faculty or staff to lead instruction |
| 4 | Beech et al, 2018 | Teaching-intensive departments must support faculty research, grant writing and publications. |
| 28 | Johnson et al, 2021 | - Small scale of program and limited number of UREG investigators can hamper a program.  - Variation in commitment to diversity, opportunities for partnerships with other entities, and faculty diversity across institutions may constrain efforts to diversify the research workforce and impact program success. |
| 46 | Rubio et al, 2019 | Junior and Senior investigators do not get protected time for research. Not enough financial support for resources. |
| 47 | Salama et al, 2021 | - Inadequate institutional support.  - When ROPS trainees transition to become faculty, they can have increasing clinical demands and increasing administrative roles that can affect scientific productivity. |
|  |  | **Implementation – Challenges** |
| 2 | Arrieta et al, 2018 | Lack of awareness among research staff about their role as mentors. |
| 28 | Johnson et al, 2021 | Common barriers to success for UREG investigators, such as lack of consistent mentorship, protected research time, and peer support. |
| 36 | Limaye et al, 2019 | DEI programs face administrative and logistical complexities when working across 3 different centers. A 2-step application process served as a barrier to some candidates. Insufficient funding was a barrier ($15,000 per scholar). Finally, coordinating times for mentors and mentees to meet was a challenge. |
| 38 | McGrew et al, 2020 | Time-consuming, frustrating, and convoluted process of getting access to VPN, databases, platforms and accounts needed for data collection. |
| 47 | Salama et al, 2021 | Inadequate infrastructure |
|  |  | **(2) Funding, resources and implementation - Opportunities** |
|  |  | **Funding Opportunities** |
| 52 | Sutton et al, 2013 | The costs for this program are low. |
| 54 | Trejo et al, 2021 | UC San Diego School of Medicine provided institutional funding for mentorship at all levels across Health Sciences, avoiding sole dependence on external funds (e.g., NIH). Office of Faculty Affairs (OFA) funded: (1) program personnel, training expenses, and evaluation (~$70,000 per year); (2) departments ($2000 each) and divisions ($500 each) for mentoring programs; and (3) senior faculty for mentorship training workshops (~$8500 for 5 faculty per year). |
| 61 | Weber-Main et al, 2020 | CAN participants achieved the highest overall award rate. This may be attributed to a combination of benefits, including awards for this career stage typically have higher rates of funding than faculty-level awards. |
|  |  | **Resources and Implementation Opportunities** |
| 1 | Abebe et al, 2019 | Program awareness and visibility. |
| 15 | Diallo et al, 2021 | Leverage and scale-up virtual infrastructure to debut as premier online professional development program for underrepresented minority researchers. |
| 28 | Johnson et al, 2021 | Strategies to increase underrepresented racial and ethnic groups investigators included: targeted outreach to these investigators, structured assistance for investigators with preparing their applications, and a KL2 program structure. |
| 53 | Thorpe et al, 2020 | Between 2008- 2017, TCHD STAR participants gar­nered grant awards in excess of $6 mil­lion, warranting national attention as a best practice in grant writing and professional development for post­doctoral fellows and junior faculty. |
| 56 | Velasquez et al, 2019 | - MHIMP learned it must increase the quantity and duration of intramural grants and to increase the eligibility period.  - The program can expand to include other underrepresented populations beyond underrepresented minority researchers to include sexual and gender minorities and investigators with disabilities. |
| 61 | Weber-Main et al, 2020 | CAN participants achieved the highest overall award rate. This may be attributed to a combination of benefits, including: (1) exceptional candidate pool (the highest number of postdoctoral fellows,) and curriculum. |
|  |  | **(3) Community Engagement and Cross-Institutional Partnership** |
|  |  | **Community Engagement and Cross-Institutional Partnership** **- Challenges** |
| 13 | Cruz et al, 2020 | Challenges included limited experience engaging with community health practitioners, complexity of working across campuses, and time pressures of completing community-engaged research within 1 year. |
| 15 | Diallo et al, 2021 | Due to the Covid pandemic, underrepresented minority mentees of one PRIDE program were unable to physically experience the community-based participatory research laboratory. As a solution, PRIDE built a virtual community-based experience. |
|  |  | **Community Engagement and Cross-Institutional Partnership** **- Opportunities** |
| 5 | Blanchard et al, 2019 | Building the Network of Minority Health Research Investigators: A Novel Program to Enhance Leadership and Success of Underrepresented Minorities in Biomedical Research. |
| 14 | Davis et al, 2020 | Society for Research on Nicotine and Tobacco Health Disparities Network's Scholarship on Professional Development of Its Recipients. |
| 17 | Estape et al, 2018 | University of Puerto Rico, Medical Sciences Campus, a Hispanic public academic health center, partners with Morehouse School of Medicine, a historically black institution, to increase workforce diversity and the capacity to address the research-practice translational gap. |
| 28 | Johnson et al, 2021 | Strategies to increase underrepresented racial and ethnic groups (UREG) investigators included: partnerships with other institutional entities. |
| 36 | Limaye et al, 2019 | Three centers for AIDS research in the Mid-Atlantic region (Johns Hopkins University, University of Pennsylvania, and the District of Columbia) developed a Scholars program for diverse researchers studying the HIV epidemic in the region. The program provided cross-institutional mentoring and training. |
| 41 | Ofili et al, 2021 | The U.S. Department of Health and Human Services (DHH) established the Research Centers in Minority Institutions (RCMI) program to develop­ biomedical research infrastructure at minority-serving institutions. RCMI developed a common evaluation process to track the inter-linked goals of workforce diversity and health equity and created a centralized database to support research collaboration. |
| 56 | Velasquez et al, 2019 | The program should continue to foster collaborations within the ACTG, expand to other HIV/AIDS clinical trials networks, and integrate with existing career development programs that target minorities at different stages of their career development. |
| 58 | Vishwanatha et al, 2018 | By integrating faculty and commu­nity stakeholders into the fellowship, we provide a co-learning experience so that faculty become exposed to CBPR. |
|  |  | **DEI Program Practices** |
|  |  | **(4) Mentoring - Challenges** |
| 24 | Harawa et al, 2017 | Many mentors are not from URGs making it more challenging for mentees to identify and socially connect with mentors. |
| 36 | Limaye et al, 2019 | Challenges to establishing a mentoring program included: heavy administrative responsibilities and logistics, which amplified complexity across 3 mentoring institutions; a burdensome two-stage application process for mentees; difficulty identifying a study where mentees could contribute, and insufficient funding per mentee ($15,000) to support research within the program. |
| 51 | Sorkness et al, 2017 | Reduced access to mentoring by URM researchers contributes to their reduced success in securing NIH funding, as demonstrated by numerous studies. |
|  |  | **(4) Mentoring - Opportunities** |
| 29 | Johnson et al, 2015 | Mentorship training for senior researchers is rare. A workshop to “Mentor the Mentors” trained mid-career and senior U.S. HIV investigators in tools and techniques specific to mentoring diverse early-stage investigators. It focused on enhancing mentoring techniques in general (definitions and mentoring process, communication strategies, consistent use of individual development plans, setting goals and expectations for the mentor-mentee relationship, time-management for mentor and mentee, work-life balance, mentor and mentee evaluation tools, leadership styles etc.), as well as topics specifically related to diversity (unconscious bias, microaggressions, diversity supplements to NIH-sponsored grants and other minority-focused funding opportunities, resiliency, and self-awareness), via didactic presentations, break-out sessions, role-playing and small-group brainstorming sessions. |
| 40 | Milburn et al, 2019 | -Mentees wanted more individual time with their mentors so institute agendas were adjusted to include at least one hour of one-on-one, in-person mentoring time at each Institute. -Mentees wanted to learn more about the mentors’ career paths, obstacles, successes, lessons learned, so mentors began sharing their respective stories over dinner each year. This helped to foster more personal connections amongst the mentees and mentors. -Mentees wished for more guidance in preparing for career development awards (e.g., K01s), so the institute had expert guest faculty and HA-STTP Scholars discuss their experiences of applying for and receiving these awards. |
| 51 | Sorkness et al, 2017 | Reduced access to mentoring by URM researchers contributes to their reduced success in securing NIH funding, as demonstrated by numerous studies. In response, NIH established the National Research Mentoring Network to offer in-person training for mentors and mentees at institutions, regional training, or national meetings, as well as via virtual platforms to expand access, scale and sustainability of DEI in HSR institutions. |
| 55 | Varkey et al, 2012 | Peer mentoring groups among women faculty in the Department of Medicine at Mayo Clinic (19 mentees across 5 peer groups) engaged through: a 1-day orientation workshop to agree on goals and processes for the 1-year program; groups with facilitators held bi-weekly or monthly mentoring meetings to develop a manuscript; and provided self-assessments and a program assessment at end of program. |
|  |  | **(5) Skills training - Challenges** |
| 3 | Awad et al, 2022 | Challenging to create course material that "could be used by a broad audience with varying levels of technical experience and diverse lived experiences” (p. 7) |
| 13 | Cruz et al, 2020 | Lack of qualified applicants; lack of grant writing skills; limited number of URM mentors; difficult for PIs to devote time for research; difficulty integrating pilot research into the TREE center activities. |
| 38 | McGrew et al, 2020 | Training length was too long due to poor pedagogy, e.g., required trainings were too time-intensive, and due to language barriers. Training complexity was introduced when CITI trainings on phlebotomy were not available in Spanish. The trainings thus required live interpreters and their translation equipment malfunctioned. |
| 46 | Rubio et al, 2019 | Lack of systematic training leads to greater demand for mentorship. The “informal curriculum” of skills training includes: grantsmanship, the administration of funded grants, management of a laboratory or research team, time management, ability to conduct research, and to write and present papers. |
| 47 | Salama et al, 2021 | Inadequate curriculum and mentorship. |
| 57 | Vermund et al, 2018 | Improve the scholars’ abilities to engage new research protocols that are developed within the network. |
|  |  | **(5) Skills training - Opportunities** |
| 18 | Fabris et al, 2016 | Program mentees have formed a support network among underrepresented colleagues. |
| 25 | Ingram et al, 2021 | Requiring pilot project applicants to communicate with Analysis Core members before application submission tremendously strengthened the quality of the applications. The plan is to increase scientists engaging and learning from each other through formal seminars and more informal gatherings. |
| 27 | Jean-Louis et al, 2016 | Mentees felt that PRIDE helped enhance their skills such as grant writing, networking, publishing, identifying mentors, presentation skills, developing research agendas, and long-term career plans. |
| 58 | Vishwanatha et al, 2018 | -The STAR Fellowship tailors proficiencies in grant writing depending on a fellow’s career stage and prior experiences.  - Conducting health disparity research requires skills to identify and implement the various domains of health disparity models (e.g., individual, social behavioral, community, policy) into their com­munity-based research programs. |
| 61 | Weber-Main et al, 2020 | - In the SETH Program, inability to assess trainees’ readiness to write was impetus to establish a screening process (semi-structured interview protocol) and an online support community. These significantly reduced participants’ time to proposal submission.  - CAN participants achieved the highest overall award rate. This may be attributed to a combination of (1) exceptional candidate pool (CAN had the highest number of postdoctoral fellows, and awards for this career stage typically have higher rates of funding than faculty-level awards) and (2) tailored program design (after the kickoff, CAN coaching sessions were held in-person on local campuses, allowing the input of multidisciplinary coaches on participants’ proposals) |
|  |  | **(6) Social network facilitation - Challenges** |
| 5 | Blanchard et al, 2019 | Lack of diversity in the biomedi­cal research workforce compromises participation of URMs in clinical re­search. NIH’s National Institute of Diabetes and Digestive and Kidney Diseases (NIDDK) launched the Network of Minority Health Research Investigators (NMRI) to address this lack of diversity among researchers and promote minority health research. |
| 11 | Campbell et al, 2013 | URG junior faculty from teaching-intensive, minority-serving institutions often lack the time, training and support to build professional networks. The Minorities Affairs Committee (MAC) of the American Society for Cell Biology (ASCB) developed the VP Program to meet the scientific needs of faculty members at minority-serving institutions by providing research training and access to professional networks to enhance scholarly practices to strengthening educational and research activities at their home institutions. |
|  |  | **(6) Social network facilitation- Opportunities** |
| 1 | Abebe et al, 2019 | The University of Pittsburgh’s Career Education and Enhancement for Health Care Research Diversity (CEED) program for Clinical Research Education which offered networking opportunities. CEED Scholars worked with a diverse and multidisciplinary group of senior faculty members and networked with both clinical and translational researchers from across the university. At the start of the year, CEED Scholars attended an informal meet-and-greet with CEED program directors, executive committee, mentors and alumni. At the end of the year, CEED Scholars hosted a poster presentation of their work to mentors and the executive committee. |
| 14 | Davis et al, 2020 | The Society for Research on Nicotine and Tobacco (SRNT) Health Disparities Network has funded a travel scholarship to promote inclusion, professional development, and diversity among investigators interested in tobacco-related health disparities research. Priority is given to applicants from US minority racial/ethnic groups, with disabilities, from sexual or gender minority groups, or from disadvantaged groups. The scholarship provides full financial support for early career investigators to attend the SRNT Annual Meeting and a 1-year membership. |
